# Implications of Chitinase 3-like 1 Protein in the Pathogenesis of Multiple Sclerosis in Autopsied Brains and a Murine Model

**DOI:** 10.3390/ijms26094160

**Published:** 2025-04-27

**Authors:** Yoshio Bando, Yasuhiro Suzuki, Chisato Murakami, Takashi Kimura, Osamu Yahara

**Affiliations:** 1 Department of Anatomy, Akita University Graduate School of Medicine, 1-1-1 Hondo, Akita 010-8543, Akita, Japan; 2 Department of Functional Anatomy and Neuroscience, Asahikawa Medical University, 2-1-1-1 Midorigaoka Higashi, Asahikawa 078-8510, Hokkaido, Japan; 3 Department of Clinical Research, National Hospital Organization, Asahikawa Medical Center, 7 Hanasakichiyo, Asahikawa 070-8644, Hokkaido, Japan; 4 Department of Neurology, National Hospital Organization, Asahikawa Medical Center, 7 Hanasakichiyo, Asahikawa 070-8644, Hokkaido, Japan

**Keywords:** multiple sclerosis, chitinase 3-like protein 1, oligodendrocyte, 2D-fluorescence difference gel electrophoresis, experimental autoimmune encephalomyelitis

## Abstract

Chitinase-3-like protein 1 (CHI3L1) has been implicated in multiple sclerosis (MS) pathology, yet its precise role remains unclear. To elucidate its involvement, we performed proteomic analysis of cerebrospinal fluid (CSF) from relapsing-remitting MS (RRMS) patients using two-dimensional difference gel electrophoresis (2D-DIGE). CHI3L1 emerged as the most upregulated protein in recurrent RRMS. ELISA confirmed significantly elevated CHI3L1 levels in recurrent RRMS and secondary progressive MS (SPMS) patients, with levels decreasing in steroid responders but increasing in non-responders. Immunohistochemistry of MS brain autopsies revealed CHI3L1 expression predominantly in mature oligodendrocytes. In an experimental autoimmune encephalomyelitis (EAE) model, CHI3L1 was highly expressed in the spinal cord, particularly in oligodendrocytes and microglia/macrophages. Functional studies demonstrated that recombinant CHI3L1 (rCHI3L1) protected oligodendrocytes from LPC-induced cell death by attenuating ER stress (GRP78, ORP150). Moreover, rCHI3L1 counteracted IFN-β- and PSL-mediated inhibition of oligodendrocyte differentiation. In microglia, rCHI3L1 suppressed LPS-induced proinflammatory markers (IL-1β, iNOS). In vivo, rCHI3L1 administration significantly mitigated EAE severity by reducing gliosis, demyelination, and axonal degeneration. These findings highlight CHI3L1 as a critical modulator of neuroinflammation and oligodendrocyte survival, positioning it as a promising therapeutic target for MS.

## 1. Introduction

Multiple sclerosis (MS) is a chronic autoimmune inflammatory disease characterized by demyelination of the central nervous system (CNS) [1,2]. Diagnosis primarily relies on clinical presentation and MRI findings, which typically reveal disseminated demyelinating lesions throughout the CNS [3,4]. Despite significant progress in understanding MS pathophysiology, a reliable biomarker for disease activity and a curative therapy remain elusive [5,6]. Therefore, considerable efforts have been directed toward identifying molecular markers that could aid in diagnosis, prognosis, and therapeutic monitoring.

Proteomic analyses using serum or cerebrospinal fluid (CSF) from MS patients have emerged as powerful tools to uncover novel disease-related proteins [7,8,9]. These approaches are particularly promising in neurological diseases, where accessible and disease-specific biomarkers are limited. However, the identification of robust and clinically relevant target proteins remains a major challenge.

To address this gap, we employed two-dimensional fluorescence difference gel electrophoresis (2D-DIGE) combined with shotgun proteomic analysis via LC-MS/MS [10]. 2D-DIGE is a sensitive gel-based method that allows for quantitative comparison of protein expression and provides insights into disease-related changes, including post-translational modifications. Using this approach, we analyzed the CSF proteomic profiles of Japanese patients with relapsing-remitting MS (RRMS). Among the proteins identified, we focused on chitinase-3-like protein 1 (CHI3L1, also known as YKL-40), which emerged as a potential key molecule involved in MS pathology.

CHI3L1 is a secreted glycoprotein with a molecular weight of approximately 40 kDa and has been previously implicated in neuroinflammatory and neurodegenerative processes [11,12,13,14,15]. Accumulating evidence suggests that CHI3L1 is produced by activated astrocytes and microglia and contributes to the regulation of immune responses within the CNS. Elevated levels of CHI3L1 in the CSF have been reported in MS patients compared to healthy controls, and increased CHI3L1 expression has been associated with the transition from clinically isolated syndrome (CIS) to definite MS [9,16,17,18,19,20,21]. Moreover, CHI3L1 has been linked to low-grade, non-lymphocytic inflammation and active neurodegeneration, particularly in progressive forms of MS [22]. These findings indicate that CHI3L1 may serve as a valuable biomarker reflecting disease activity, progression, and possibly therapeutic response.

Given these insights, we aimed to elucidate the functional role of CHI3L1 in MS-related neuroinflammation. In this study, we performed both in vitro and in vivo experiments to investigate how CHI3L1 contributes to disease pathogenesis, with the ultimate goal of clarifying its potential as a biomarker and therapeutic target in MS.

## 2. Results

### 2.1. Proteomic Analysis in CSF of RRMS Patients

**Figure 1:**
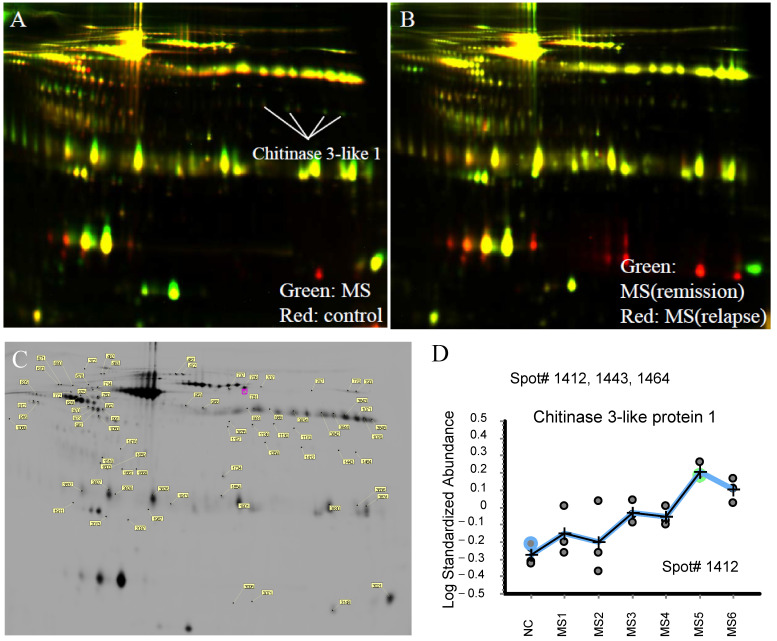
Isolation of CHI3L1 by 2D-DIGE analysis. CSF from either normal control (NC group, NC-1-3), MS patient (treated group, MS-2,4,6), MS patient (remission; MS-5), or MS patient (relapse; MS−1,3) was subjected to 2D-DIGE followed by LC-MS/MS. (**A**) Proteins in the CSF from both the treated MS patient (MS−6, green) and the normal control (NC-1, Red) were separated by 2D-DIGE. A representative gel image is shown. (**B**) Proteins in the CSF from both the remissioned MS patient (MS-5, green) and the relapsed MS patient (MS-6, Red) were separated by 2D-DIGE. A representative gel image is shown. (**C**) Isolated spots by 2D-DIGE are shown. Spots #1412, 1443, and 1464 were CHI3L1. (**D**) The ratio of CHI3L1 expression (relative to NC) in the CSF of MS patients is shown (Spot#1412). The vertical axis indicates Log standardized abundance. Representative data is shown.

Information on the CSF samples used in this study is presented in Table 1. The state of all RRMS patients was the recurrent phase at the time of the diagnosis. Two-dimensional difference gel electrophoresis (2D-DIGE) of CSF samples from RRMS patients revealed disease state-dependent alterations in peptide composition (Figure 1). Comparative analysis of CSF samples before and after treatment identified 668 peptides with a *p*-value below 0.01 in a paired *t*-test. Among the identified proteins, six were highly ranked, with CHI3L1 being the most significantly upregulated (Figure 1C). Orbitrap mass spectrometry confirmed an increased level of CHI3L1 expression in post-treatment CSF samples (Figure 1D). Notably, CHI3L1 expression exhibited dynamic changes corresponding to disease progression.

### 2.2. Upregulation of CHI3L1 in the CSF of Recurrent RRMS Patients by ELISA

**Figure 2:**
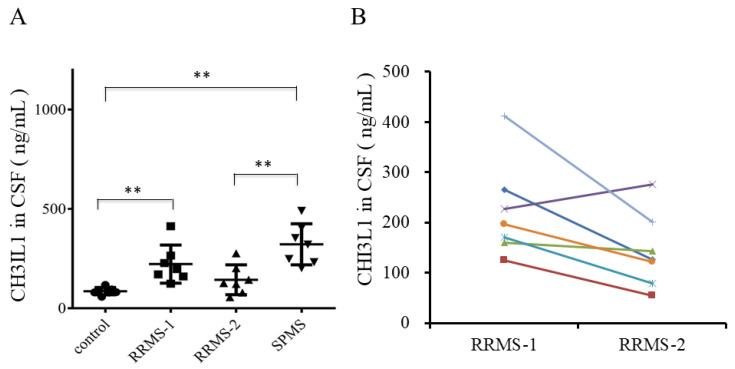
The expression of CHI3L1 in the CSF from MS patients. (**A**) The expression level of CHI3L1 in the CSF from either normal control (NC) or MS patients (RRMS-1: recurrence, RRMS-2: remission, SPMS: secondary progressive) is confirmed by ELISA. ** *p* < 0.05 (Mann–Whitney U test). (**B**) CHI3L1 expression during the CSF in recurrence (RRMS-1) and remission (RRMS-2). Solid lines indicate identical patients. Note that CHI3L1 is reduced in patients who have responded to treatment with steroids, except for one patient shown in solid purple line.

CHI3L1 levels in CSF from healthy controls (control), RRMS patients (RRMS-1: recurrence, RRMS-2: remission), and SPMS patients were examined via ELISA. CHI3L1 expression was significantly elevated in the CSF of seven recurrent RRMS patients compared to healthy controls (*p* = 0.0006, Figure 2A). Although CHI3L1 expression was higher in recurrent RRMS than in remission RRMS, the latter showed no significant difference from healthy controls. Conversely, SPMS patients exhibited the highest CHI3L1 expression, surpassing both healthy subjects (*p* = 0.0006) and remission RRMS patients (*p* = 0.0041). Further, CSF CHI3L1 levels declined in steroid pulse therapy responders but increased in non-responders (Figure 2B).

### 2.3. CHI3L1 Expression in Oligodendrocytes in MS Autopsied Brain Tissue

**Figure 3:**
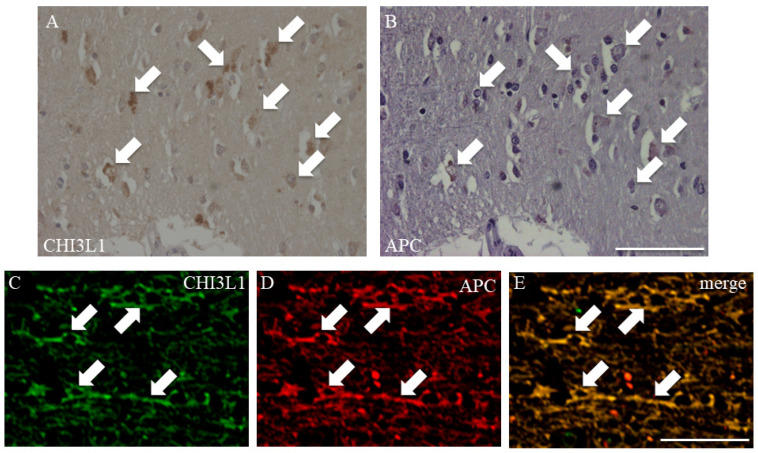
Immunohistochemistry investigation in human autopsied MS brain. Immunohistochemistry for CHI3L1 was performed on serial paraffin sections of autopsy brains of human MS patients. The sections were stained with either anti-CHI3L1 antibody (**A**) or anti-APC antibody (**B**), a marker for mature oligodendrocytes. CHI3L1 is co-stained in APC-positive oligodendrocytes (Arrows). (**C**–**E**) CHI3L1 expression in human autopsy specimens was also examined using fluorescent double staining. Each panel shows CHI3L1 ((**C**), green), APC ((**D**), red), and merge (**E**). Similar to (**A**,**B**), CHI3L1 is co-stained in APC-positive oligodendrocytes (Arrows). Scar bar = 50 μm.

To determine CHI3L1-expressing cell types, immunohistochemistry was performed on autopsied brain sections from RRMS patients. As previously reported, CHI3L1 was detected in the cell bodies of some astrocytes (Supplement Figure S1, white arrows), while its expression in astrocytic processes was minimal (Supplement Figure S1A,B). In contrast, we observed CHI3L1 immunoreactivity that strongly colocalized with APC, indicating for the first time that mature oligodendrocytes express CHI3L1 in and around MS plaques (Figure 3A,B, arrows). Double immunofluorescence confirmed CHI3L1 expression in mature oligodendrocytes around MS plaques (Figure 3C,D, arrows). However, CHI3L1 expression was not detected within the MS plaques themselves. These results highlight a previously underexplored function of oligodendrocytes and suggest that their contribution to MS pathogenesis, particularly through CHI3L1-related pathways, may warrant further investigation.

### 2.4. Elevated CHI3L1 Expression in Oligodendrocytes and Macrophages/Microglia in the EAE Spinal Cord

**Figure 4:**
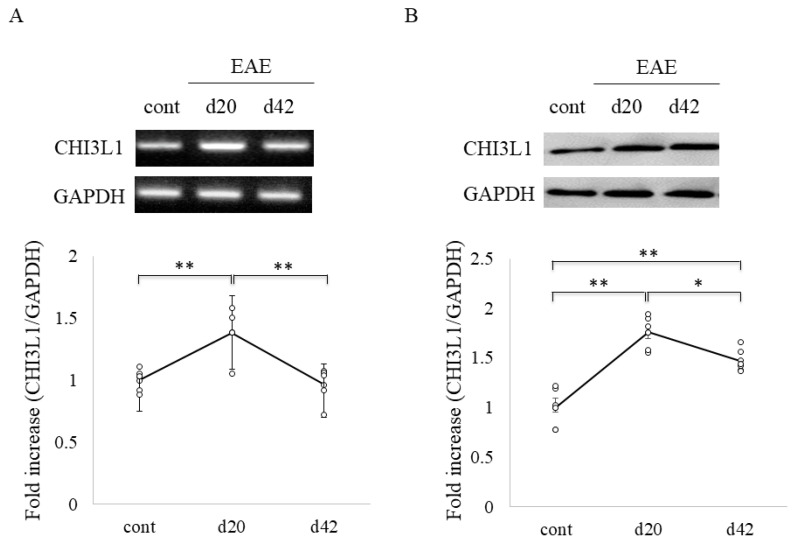
CHI3L1 expression in EAE. CHI3L1 expression in the spinal cord of EAE mice was examined by RT-PCR (**A**) and immunoblot (**B**) (*n* = 6). GAPDH was used as a loading control. Quantitative analysis is also shown. * *p* < 0.05, ** *p* < 0.01.

**Figure 5:**
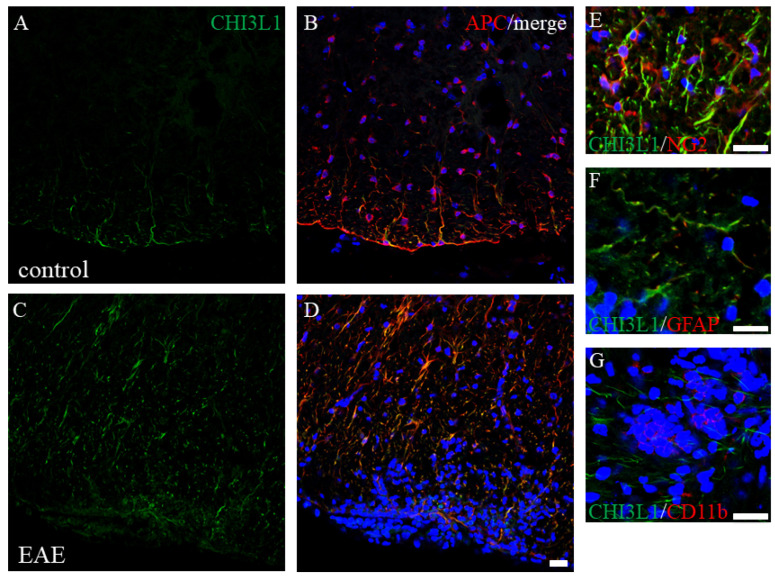
CHI3L1 is expressed in oligodendrocytes of the spinal cord in EAE mice. Representative images in Naïve (**A**,**B**) and EAE mice (**C**–**G**) at the peak disease stage (day 20) after MOG immunization are shown. Frozen sections of the spinal cord were stained with anti-CHI3L1 antibodies ((**A**,**C**,**E**–**G**), green). These sections were also immunostained with either anti-APC antibody (a marker for mature oligodendrocyte) ((**B**,**D**), red), anti-NG2 antibody (a marker for oligodendroglial progenitor cells) ((**E**), red), anti-GFAP antibody (a marker for astrocytes) ((**F**), red), or anti-CD11b antibody (a marker for macrophage/microglia) ((**G**), red). DAPI staining was also performed (blue). Merged images are shown (**B**,**D**–**G**). (*n* = 6). Scar bar = 20 μm.

To assess CHI3L1 expression under neuroinflammatory conditions, we examined MOG-induced EAE mice, a model of MS. RT-PCR analysis revealed CHI3L1 expression in control mouse spinal cords, with significantly increased levels in EAE mice (Figure 4A). This was corroborated by immunoblot analysis (Figure 4B). Time-course analysis indicated that CHI3L1 expression peaked during acute disease and declined in the chronic phase (Figure 4). Double immunofluorescence studies further demonstrated that CHI3L1 expression was markedly elevated in EAE white matter (Figure 5C,D) compared to controls (Figure 5A,B). CHI3L1 was predominantly expressed in APC-positive oligodendrocytes and CD11b-positive macrophages/microglia (Figure 5G), with minimal expression in NG2-positive oligodendrocyte progenitor cells and GFAP-positive astrocytes (Figure 5E,F).

### 2.5. Protective Effects of rCHI3L1 Against LPC-Induced Cell Death in FBD-102b Cells

**Figure 6:**
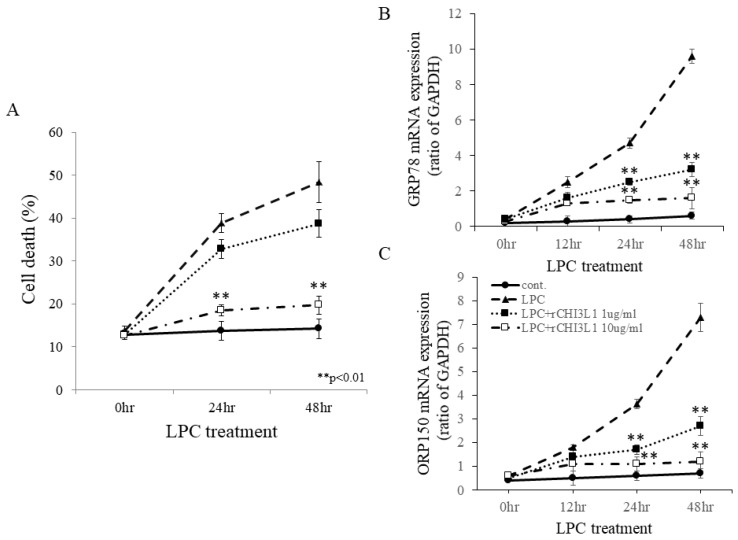
Effects of rCHI3L1 on LPC-induced oligodendroglial cell death in FBD-102b cells. (**A**) To induce oligodendroglial cell death, FBD-102b cells, an oligodendroglial cell line, were exposed to LPC for up to 48 h (solid line + closed circle: control (mock), thick dashed line + closed triangle: LPC, dotted line + closed square: rCHI3L1 1 μg/mL, single-pointed line + open square: rCHI3L1 10 μg/mL) (*n* = 6). Expression of ER-resident molecular chaperone, GRP78/Bip (**B**) and ORP150 (**C**) in LPC-treated FBD-102b cells by RT-PCR are shown (solid line + closed circle: control (mock), thick dashed line + closed triangle: LPC, dotted line + closed square: rCHI3L1 1 μg/mL, single-pointed line + open square: rCHI3L1 10 μg/mL) (*n* = 6). The graph is expressed as a ratio to GAPDH. ** *p* < 0.01.

**Figure 7:**
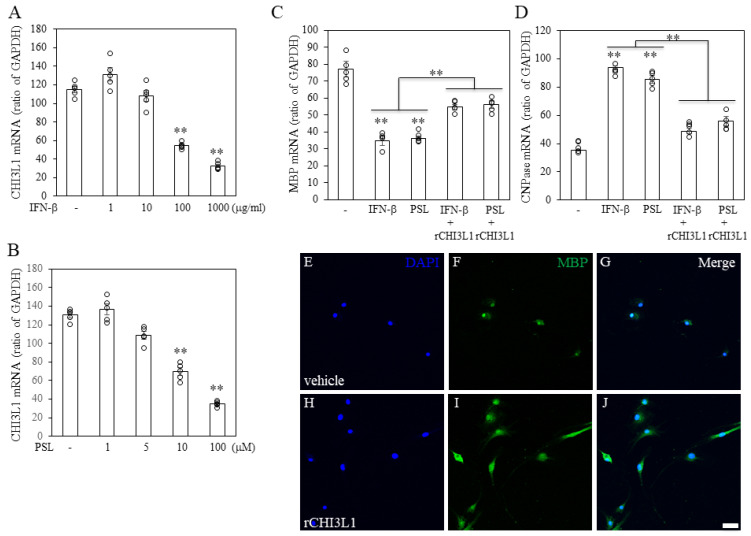
Effect of rCHI3L1 on oligodendrogenesis in FBD-102b cells. Expression of CHI3L1 in FBD-102b cells treated with either IFN-β (**A**) or PSL (**B**) was analyzed by RT-PCR. (**C**,**D**) Expression of MBP in FBD-102b cells treated with either non-treat (vehicle, −), IFN-β, PSL, IFN-β+rCHI3L1 or PSL+rCHI3L1 was analyzed by RT-PCR. These graphs are expressed as a ratio to GAPDH. (*n* = 5). (**E**–**J**) Immunocytochemistry for MBP was performed in FBD-102b cells treated with either vehicle (**E**–**G**) or rCHI3L1 (**H**–**J**). ** *p* < 0.01. MBP expression ((**F**,**I**), green) was increased by treatment with rCHI3L1. FBD-102b cells were also stained with DAPI ((**E**,**H**), blue). Merged images are shown (**G**,**J**), (*n* = 6), Scar bar = 20 μm.

To explore CHI3L1′s effects on oligodendrocytes and microglia/macrophages, recombinant CHI3L1 (rCHI3L1) was added to cultures of FBD-102b oligodendroglial cells and BV2 microglial cells stimulated with LPC. High concentrations of rCHI3L1 significantly suppressed LPC-induced oligodendroglial cell death in FBD-102b cells (Figure 6A) and ES-derived oligodendrocytes. LPC-induced ER stress was indicated by the upregulation of GRP78 and ORP150, two ER-resident molecular chaperones (Figure 6B,C). Notably, rCHI3L1 suppressed LPC-induced GRP78 and ORP150 expression, suggesting a role in ER stress attenuation and oligodendrocyte survival. Further, RT-PCR analysis showed that IFN-β and PSL downregulated CHI3L1 expression in a dose-dependent manner (Figure 7A,B). Since IFN-β and PSL have been reported to suppress oligodendrocyte differentiation, their effects on differentiation were examined. IFN-β and PSL treatment reduced MBP expression (Figure 7C) while increasing CNPase expression, indicative of immature oligodendrocytes (Figure 7D). However, rCHI3L1 treatment rescued MBP expression, promoting oligodendrocyte differentiation even in the presence of IFN-β or PSL (Figure 7E–J). These findings suggest that CHI3L1 plays a crucial role in oligodendrocyte maturation.

### 2.6. Attenuation of Microglial Activation by rCHI3L1

**Figure 8:**
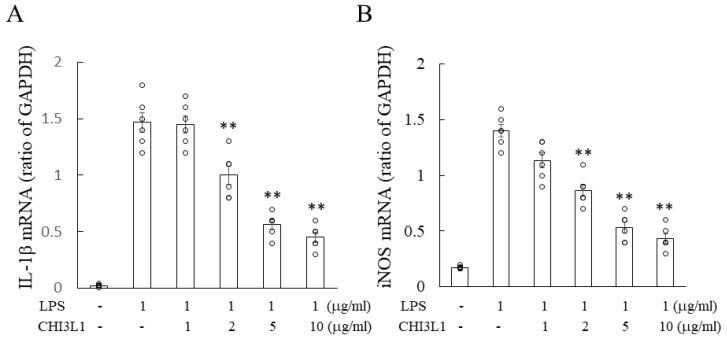
Effect of rCHI3L1 on inflammatory cytokine production in BV2 cells. Expression of IL-1β (**A**) and iNOS (**B**) in LPS-treated BV2 cells treated with either IFN-β (**A**) or PSL (**B**) was analyzed by RT-PCR (*n* = 6). ** *p* < 0.01.

To evaluate CHI3L1′s anti-inflammatory effects, BV2 microglial cells were stimulated with LPS in the presence or absence of rCHI3L1. RT-PCR analysis showed that LPS significantly increased IL-1β and iNOS mRNA expression, while rCHI3L1 treatment attenuated this response, suggesting its potential role in suppressing neuroinflammation (Figure 8).

### 2.7. Effects of rCHI3L1 on EAE Development

**Figure 9:**
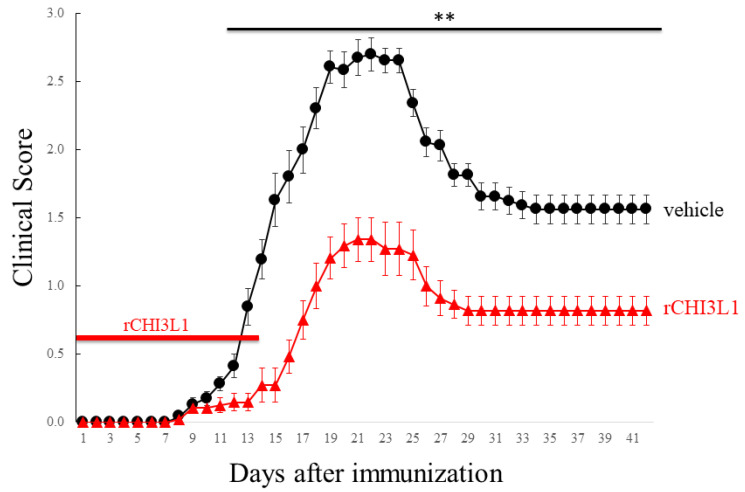
Amelioration of MOG-induced EAE pathology by rCHI3L1. Progression of EAE was daily monitored and scored as disease severity on a clinical scale (vehicle, closed circle shown in black; closed triangle shown in red; rCHI3L1). Mice were treated with rCHI3L1 for 2 weeks (days 0–14 after immunization, red bar). (*n* = 10) ** *p* < 0.01.

**Figure 10:**
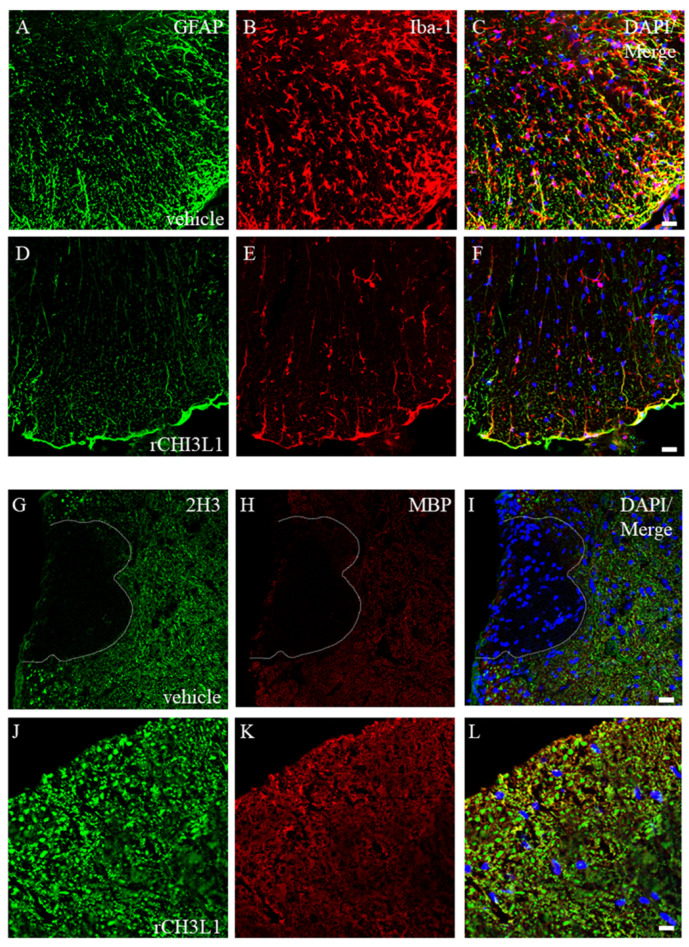
Effect of rCHI3L1 on EAE-induced gliosis in vivo. Vehicle- (**A**–**C**,**G**–**I**) and rCHI3L1-treated (**D**–**F**,**J**–**L**) EAE mice were perfused at the peak disease stage after MOG immunization. Frozen sections of the spinal cord were stained with either anti-GFAP antibody (**A**,**D**) anti-Iba-1 antibody (**B**,**E**), anti-2H3 antibody (a marker for neurofilament in the axon) (**G**,**J**), or MBP (a marker for myelin) (**H**,**K**). DAPI staining (blue) was also performed (*n* = 6). Merged images are also shown (**C**,**F**,**I**,**L**). (**C**–**I**) The dotted line shows an EAE-induced demyelinated lesion. Scar bar = 20 μm.

To investigate rCHI3L1′s impact on EAE progression, it was administered intraperitoneally (i.p) every other day from day 0 to day 14 in EAE-induced mice. rCHI3L1-treated mice exhibited significantly reduced EAE incidence (Figure 9). Immunohistochemistry revealed suppressed gliosis, as evidenced by reduced GFAP-positive astrocytes and Iba-1-positive microglia in rCHI3L1-treated mice (Figure 10D–F) compared to controls (Figure 10A–C). Furthermore, neurofilament (2H3) and myelin (MBP) intensities were preserved in rCHI3L1-treated mice but markedly reduced in controls (Figure 10G–L). These findings indicate that rCHI3L1 mitigates demyelination and axonal degeneration in EAE, supporting its potential as a therapeutic target in MS.

## 3. Discussion

This study provides compelling evidence for the role of CHI3L1 in MS, particularly through its expression in CSF, oligodendrocytes, and microglia/macrophages, which is tightly linked to MS pathophysiology. Elevated CHI3L1 levels were observed in the CSF of both RRMS and SPMS patients, with a decrease following methylprednisolone treatment, suggesting its involvement in inflammation. These findings corroborate earlier reports that associate CHI3L1 with MS prognosis and disability progression [9,18,19,20,21]. Histologically, our study provides novel evidence of CHI3L1 expression in oligodendrocytes, an observation previously unreported in human CNS tissue. This highlights its relevance in MS pathology and suggests that CHI3L1 may be expressed not only in astrocytes but also in oligodendrocytes and microglia. While earlier studies have emphasized CHI3L1 expression in astrocytes [22,23,24,25], our findings, based on autopsy brain samples and the EAE model, indicate that CHI3L1 expression is more prominent in oligodendrocytes and microglia than in astrocytes (Figure 3 and Figure 5). This discrepancy may be due to the pathological conditions in the autopsy brain. However, considering the unexplained effects reported in CHI3L1 deletion models in astrocytes [22,23,24,25], it is likely that CHI3L1 also plays a significant role in other glial cells, affecting their functions in the context of MS. Since it was previously believed to be expressed predominantly in astrocytes, the focus had not been on other cell types such as oligodendrocytes and microglia/macrophages.

CHI3L1, a chitinase-like protein, has been implicated in various inflammatory processes [13,14,15,18,25,26,27]. Although it lacks enzymatic activity, CHI3L1 is involved in tissue remodeling and the regulation of immune responses [12,13]. Previous studies have demonstrated that CHI3L1 regulates Th2 responses [28,29,30], modulates IL-13-mediated inflammation [31,32,33], and suppresses matrix metalloproteinase (MMP) production [34]. Our findings align with these reports, showing that CHI3L1 is upregulated in microglia/macrophages and oligodendrocytes in both human (Figure 3) and animal models of MS (Figure 5), suggesting its pivotal role in the inflammatory milieu of MS.

Despite the common finding of CHI3L1 upregulation during inflammation, its functional role remains controversial [13,14,15]. The function of CHI3L1 may vary considerably depending on the experimental system, the intensity of inflammation, and the pathological microenvironment [18]. While the function of CHI3L1 in astrocytes has been well-characterized [22,23,24,25], its role in oligodendrocytes and microglia has received less attention. Our study, therefore, focused on elucidating the specific functions of CHI3L1 in these glial cells.

In the EAE model, CHI3L1 expression peaked during the acute phase of the disease and declined in the chronic stages (Figure 4), which mirrors the pattern observed in MS patients’ CSF. Immunohistochemical analysis revealed that CHI3L1 was predominantly expressed in oligodendrocytes and microglia/macrophages, with lower expression observed in astrocytes and oligodendrocyte progenitors (Figure 5). These findings support the hypothesis that CHI3L1 may contribute to the inflammatory response within the CNS, potentially influencing oligodendrocyte survival and differentiation. In vitro experiments further clarified the functional role of CHI3L1 in oligodendrocyte survival. CHI3L1 was found to protect oligodendrocytes from LPC-induced death, likely by reducing endoplasmic reticulum (ER) stress markers, such as ORP150 and GRP78 (Figure 6) [35]. These results suggest that CHI3L1 may serve as a neuroprotective factor by alleviating ER stress, which is a crucial mechanism in MS, where demyelination and neuronal damage are central to disease progression. Furthermore, CHI3L1 promoted oligodendrocyte differentiation (Figure 7), a key process for remyelination in MS. This finding is consistent with studies on CHI3L3/Ym-1, which also promote oligodendrogenesis and inhibit EAE, suggesting a broader role of CHI3L1 in CNS repair [36].

The administration of recombinant CHI3L1 (rCHI3L1) in EAE mice resulted in improved disease outcomes, including reduced severity, gliosis, demyelination, and axonal degeneration (Figure 9 and Figure 10). These results suggest that CHI3L1 not only protects oligodendrocytes but also facilitates remyelination and neuroprotection, positioning it as a promising therapeutic target for MS. Furthermore, rCHI3L1 administration reduced inflammatory cytokine production and promoted an M2 microglial phenotype, which highlights its anti-inflammatory properties (Figure 8) [37,38]. These results were confirmed in primary murine microglia, supporting the potential therapeutic application of CHI3L1 in controlling CNS inflammation. However, the role of CHI3L1 in MS is complex, as prior studies have suggested both pro-inflammatory and anti-inflammatory effects, depending on the context [13,14,15,18]. While this study emphasizes its anti-inflammatory properties in MS, further research is necessary to clarify its precise function in regulating the immune response and its therapeutic potential. CHI3L1′s role in glial cells may be context-dependent, influenced by factors such as expression levels, disease stage, and other inflammatory mediators, which warrants further investigation. A limitation of this study is that we could not assess whether CHI3L1 in astrocytes exacerbates inflammation. Further studies utilizing knockout mice lacking CHI3L1 specifically in astrocytes may clarify this issue.

Despite these challenges, the therapeutic potential of CHI3L1 in MS remains promising. Our findings suggest that targeting CHI3L1 could improve oligodendrocyte survival, promote remyelination, and reduce inflammation in MS. Additionally, modulation of CHI3L1 levels through disease-modifying therapies such as natalizumab and fingolimod [8,39], which are known to reduce CHI3L1 levels, could provide another strategy for developing effective treatments for MS. Future studies should explore the mechanisms that regulate CHI3L1 expression and its interaction with other key players in MS pathogenesis, such as T cells, microglia, and oligodendrocytes.

## 4. Materials and Methods

### 4.1. Patients

This study was approved by the Ethics Committee of Asahikawa Medical Center, National Hospital Organization. Informed consent was obtained from each participant prior to the study. Agreement for this study was made in signing the consent form (#9–21, National Hospital Organization, Asahikawa Medical Center, Asahikawa, Japan) and was conducted with the informed consent of the patients. Diagnosis of MS was made according to the McDonald criteria by two more neurologists [3]. Patient information is summarized in Table 1, respectively. AQP-4 antibody was estimated by Dr. T. Takahashi (Dept. of MS Therapeutics, Tohoku University Graduate School of Medicine, Miyagi, Japan). All enrolled MS patients were negative for the AQP-4 antibody.

To perform shotgun proteomic analysis by 2D-DIGE, three patients (3 women) with relapse-remission MS (RRMS) and 3 controls (3 men) were enrolled. All patients with RRMS were treated with 1000 mg methylprednisolone intravenously for 3 days in the relapse phase. To analyze CHI3L1 expression in CSF, seven patients with RRMS (1 man and 6 women, and the age range was from 24 to 64 years), seven patients with SPMS (1 man and 6 women, and the age range was from 45 to 61 years), and 7 healthy controls (3 men and 4 women, and the age range was from 17 to 58 years) were enrolled in this prospective study (National Hospital Organization, Asahikawa Medical Center, Asahikawa, Japan). Also, the number of steroid responders was six patients (1 man and 5 women, and the age range was from 23 to 64 years), and the number of steroid non-responders was one patient (1 woman and the age was 25 years).

### 4.2. Preparation of CSF Samples

CSF was collected both before and within 21 days with methylprednisolone. After the lumbar puncture, 8–10 mL of CSF obtained from each individual was collected in polypropylene tubes, and the fluids were centrifuged for 5 min at 3000 rpm. The supernatants were immediately transferred to new polypropylene tubes and stored at −80 °C until use as cell-free CSF.

### 4.3. Proteomics Analysis Using the CSF from MS Patients

Briefly, all CSFs were affinity-purified to achieve higher sensitivity and specificity for less abundant proteins. All samples were prepared and analyzed by 2D-DIGE in a blinded and randomized sequence, and the total protein concentration loaded was 600 μg/gel. Isoelectric focusing was performed using 24 cm IPG strips pI 3–10 (GE Healthcare, Uppsala, Sweden), and second-dimension separation was conducted using 12% SDS-PAGE by the Ettan DALT II system (GE Healthcare, Uppsala, Sweden). The 2D gels were scanned by Typhoon 9400 (GE Healthcare, Uppsala, Sweden) immediately. Image files were processed using the software Decyder-DIA (version 6.0-7.0, GE Healthcare, Uppsala, Sweden). Detected protein spots were then matched between gels, and a synthetic master image was prepared to represent a majority of the protein spots present in all gels and group samples. Protein spots of interest were excised from gels using a Spot Picker (GE Healthcare, Uppsala, Sweden), and transferred to 96-well plates. Picking each gel was digested with trypsin and analyzed using nanoLC-ESI-MS (capLC; Waters, Tokyo, Japan) and Q-Tof micro (Waters, Tokyo, Japan). This analysis was completely performed by the Chemical Evaluation and Research Institute (CERI, #937-10-P-0087, Saitama, Japan).

### 4.4. Determination of CHI3L1 Levels in CSF

CHI3L1 levels in CSF were determined using a commercially available enzyme-linked immunosorbent assay (ELISA) kit (Quantikine ELISA kit, R&D Systems, Inc., Minneapolis, MN, USA) according to the manufacturer’s recommendations. Diluted CSF samples were measured in duplicate.

### 4.5. Immunohistochemistry

The autopsied MS brains were ordered in the NICHD Brain and Tissue Bank for Developmental Disorder (NICH Contract #HHSN275200900011C, Ref. No. NO1-HD-9-0011). Tissue specimens were fixed in 4% paraformaldehyde and embedded in paraffin. Autopsy tissues were cut into 4 μm-thick sections and stained with hematoxylin-eosin (HE) and Kluver–Barrera (KB) staining for routine pathological examination. In addition, tissues were stained with an avidin-biotin technique for immunohistochemistry. After deparaffinization, intrinsic peroxidase activity was blocked by incubation with 3% H_2_O_2_ in phosphate-buffered saline (PBS) for 10 min. Immunosaver (Nissin EM, Tokyo, Japan) was used for better antigen retrieval for APC antigen, an oligodendroglial marker. The primary antibody was human CHI3L1 (R&D, AF2599, MN, USA). APC (a marker for mature oligodendrocytes, OP80, Caibiochem/Merck/Millipore, Darmstadt, Germany), visualized by secondary antibodies including anti-mouse biotinylated immunoglobulin. In some immunofluorescence staining, Alexa Fluor-conjugated secondary antibodies (Thermo Fisher Scientific, Eugene, OR, USA) were used for visualization. Alternatively, animals were sacrificed and perfused with cold PBS followed by 4% paraformaldehyde (PFA) in 0.1 M phosphate buffer (PB, pH 7.4) [40,41]. Spinal cords were removed and immersed in 30% sucrose in 0.1 M PB for 1–2 days. Spinal cords were then frozen in an OCT medium. Frozen 14 µm sections were prepared on a cryostat and then stored at −30 °C until use. In some experiments, the sections were stained with Luxol fast blue/cresyl violet (LFB/CV) to assess demyelination as described previously [40]. For immunohistochemistry, the sections were immunostained with anti-CHI3L1 antibody (AF2599, 1:1000, R&D, MN, USA), anti-APC antibody (a marker for mature oligodendrocytes, OP80, 1:1000, Calbiochem/Merck/Millipore, Darmstadt, Germany), anti-Iba 1 antibody (a marker for macrophages/microglia, 1:1000, FUJIFIRM WAKO, Osaka, Japan), anti-GFAP antibody (a marker for astrocytes, 1:1000, Sigma, St. Louis, MO, USA) or anti-2H3 antibody (a marker for axon, 1:1000, Developmental Studies Hybridoma Bank, Iowa, IA, USA), anti-MBP antibody (a marker for myelin, 1:1000, Millipore, Darmstadt, Germany) and anti-NG2 antibody (a marker for oligodendroglial progenitor cells, 1:500, Millipore, Darmstadt, Germany) [40,41,42]. Immunostaining was performed following a standard fluorescein protocol. Briefly, sections were blocked with 2% normal goat serum, 5% BSA, and 0.2% Triton X-100 and then incubated with primary antibodies at 4 °C overnight. For dual staining, Alexa-488 and Alexa-594 (Molecular Probes, Eugene, OR, USA)-conjugated secondary antibodies were used to visualize primary antibodies. The sections were analyzed with a confocal laser microscope (FV-1000D, OLYMPUS, Tokyo, Japan) with software (Fluoview, FV10-ASW 3.0, OLYMPUS, Tokyo, Japan) [40,41,42]. Each pathology group consisted of tissue sections from at least six animals.

### 4.6. Animals

All experimental protocols were carried out according to the guidelines laid down by the NIH, USA, regarding the care and use of animals for experimental procedures, and the protocols were approved by the institutional animal care and use committee of Asahikawa Medical University. Every attempt was made to minimize animal suffering and to reduce the number of mice used. Female C57BL/6 (B6) mice were obtained from The Jackson Laboratory (Sankyo Laboratory, Tokyo, Japan). EAE is an animal model for human multiple sclerosis (MS). EAE induction was performed as previously described [40,43]. In brief, the peptide MOG35-55 (MEVGWYRSPFSRVVHLYRNGK) was synthesized by Scrum (Tokyo, Japan) and purified to 99% by HPLC. B6 female mice, aged 6–8 weeks, were immunized s.c. in the flank with an emulsion of 150 μg of MOG35-55 peptide in 75 μL of phosphate-buffered saline and 75 μL of complete Freund’s adjuvant containing 0.4 mg of heat-inactivated *Mycobacterium tuberculosis* (H37Ra; Difco Laboratories, Franklin Lakes, NJ, USA). Each animal also received 200 ng of pertussis toxin (Sigma-Aldrich, St. Louis, MO, USA) through i.p. injection on days 0 and 2 postimmunization. EAE clinical score was determined in a blinded fashion as described previously [40]: 0, no disease; 1, limp tail or isolated weakness of gait without limp tail; 2, partial hind limb paralysis; 3, total hind limb or partial hind and front limb paralysis; 4, total hind leg and partial front leg paralysis; and 5, moribund or dead animal. Paralyzed mice were given easy access to food and were hand-watered at least twice daily. In the rCHI3L1 IP administration experiment, we attempted to administer recombinant protein by taking advantage of the fact that the blood-brain barrier is disrupted in the EAE model. In brief, 10 μg of rCHI3L1 (R&D, MN, USA) was administered i.p. every other day in EAE-induced mice (days 7–21 after immunization). A mean clinical score was assigned to each group using this scale and used for statistical analysis (Mann–Whitney U-test).

### 4.7. Determination of CHI3L1 Expression by RT-PCR and Western Blot Analysis

Mice were given a certain dose of anesthetic (0.3 mg/kg medetomidine (Nihon Zenyaku Kogyo, Fukushima, Japan), 4 mg/kg midazolam (Sando, Tokyo, Japan), and 5 mg/kg butorphanol (Meiji Seika Pharma, Tokyo, Japan), i.p), and the tissues were quickly dissected. Total RNA was extracted using TRIzol reagent (Invitrogen Life Technologies, Carlsbad, CA, USA). Three micrograms of total RNA in each sample were subjected to first-strand cDNA synthesis with AMV reverse transcriptase (Promega, Madison, WI, USA) at 42 °C for 1 h. Aliquots from the RT reaction were used for PCR amplification using specific primer pairs (CH3IL1-S: 5′-AGGCTTTGCGGTCCTGAT-3, CHI3L1-AS: 5′-CCAGCTGGTGAAGTAGCAGA-3′, ORP150-S: 5′-TGTCCTCTTGGCAGACCTGTTG-3′, ORP150-AS: 5′-TTTTCCTCCG AGATTCCTTGTTC-3′; GRP78-S: 5′-CCC CAG ATT GAA GTC ACC TTT GAG-3′, GRP78-AS: 5′-CAGGCGGTTTTGGTCATTG-3′, myelin basic protein (MBP)-S: 5′-CAGAAGAGACCCTCACAGCG-3′, MBP-AS: 5′-GTTTTCATCTTGGGTCCGGC-3′, CNPase (2′, 3′-cyclic nucleotide 3′- phosphodiesterase) -S: 5′-CTGGGGAATCACAAGGCCTT-3′, CNPase-AS: 5′-CGTGAAGATGGCCTTGACCT-3′, GAPDH-S: 5′-CGGGAAGCCCATCACCATCA-3′, GAPDH-AS: 5′-GAGGGGCCATCCACAGTCTT-3′) (Sigma/Merck, Tokyo, Japan) [44,45]. The reaction products were electrophoretically separated on a 1–2% agarose gel and visualized by staining with ethidium bromide. For Western blot analysis, tissues were lysed in RIPA buffer (1% Nonidet P-40, PMSF, EDTA in PBS) as previously reported [40]. After determination of the protein concentration (DC protein assay kit; BioRad, Hercules, CA, USA), 10–20 µg of protein extract was separated by 10–15% sodium dodecyl sulfate-polyacrylamide gel electrophoresis (SDS-PAGE), transferred to PVDF paper (Millipore, Bedford, MA, USA), and immunostained with either anti-CH3IL1 antibody (1:1000, R&D, MN, USA) or anti-GAPDH antibody (1:1000, Sigma-Aldrich, St. Louis, MO, USA). For detection, HRP-conjugated secondary antibodies (1:1000, GE Healthcare Uppsala, Sweden) were used, followed by ECL chemiluminescence development (GE Healthcare Uppsala, Sweden) with a lumino image analyzer (LAS-3000; Fuji, Tokyo, Japan).

### 4.8. Cell Culture

Mouse oligodendroglial progenitor cell line, FBD-102b cells derived from p53-/- fetal brain (kindly obtained from Dr. Tomooka [46]) were cultured in DMEM containing 10% FCS at 37 °C in a 5% CO_2_ incubator. To induce oligodendroglial differentiation, cells were cultured for 4 days in DMEM containing 0.5% FCS or serum-free medium (N2; Gibco/Thermo Fisher Scientific, Eugen, OR, USA) supplemented with 3 × 10^−8^ M triiodothyronine (T3; Sigma, St. Louis, MO, USA) and 1 ng/mL biotin (Sigma, St. Louis, MO, USA) [43]. In some experiments, differentiated cells were then treated with 100 nM l-α-Lysophosphatidylcholine (lysolecitin/LPC; Sigma, St. Louis, MO, USA) for up to 48 h to induce cell death. For IFN-β or prednisolone (PSL) treatment, undifferentiated FBD-102b cells were treated with either IFN-β (1–1000 μg/mL, Sigma, St. Louis, MO, USA) or PSL (1–100 μM, Sigma, St. Louis, MO, USA) for 48 h. At the same time, in some experiments, recombinant CH3IL1 (rCHI3L1; 1 μg/mL or 10 μg/mL; R&D, MN, USA) was also added into the culture medium. Cell death was monitored by LDH assay kit (Kyokuto, Tokyo, Japan) and TUNEL staining Apoptotic/Necrotic/Healthy Cells Detection Kit (PromoKine, Heidelberg, Germany). In addition, mouse embryonic stem (ES)-derived oligodendrocyte progenitor cells (OPCs) were used in some experiments [40]. ES-derived OPCs plated onto poly-L-lysine-coated dishes (BD Biosciences, San Jose, CA, USA) were stimulated with 30 ng/mL triiodothyronine (T3; Sigma, St. Louis, MO, USA) for 7 days to their maturation. Since the results were basically similar, the results for the FBD-102b cells are only shown as representative unless otherwise noted. BV2 cells, an immortalized murine microglial cell line, were cultured in DMEM supplemented with 10% FCS. BV2 cells were stimulated with 1 μg/mL lipopolysaccharide (LPS) (Sigma, St. Louis, MO, USA) [43].

### 4.9. Statistical Analysis

For statistical analysis, the Mann–Whitney U test was performed. All tests were classified as significant if the *p* value was less than 0.05. PRISM was also used for these analyses. The data represent the mean ± SEM.

## 5. Conclusions

In conclusion, CHI3L1 is a critical regulator in the pathogenesis of MS, with roles in inflammation, oligodendrocyte survival, differentiation, and remyelination. The findings presented here highlight the potential of CHI3L1 as a novel biomarker for MS prognosis and a promising therapeutic target. Targeting CHI3L1 could provide new strategies for promoting CNS repair and managing MS progression, offering hope for improved treatments in the future.

## Figures and Tables

**Table 1:** Clinical characteristics of three recurrent MS patients and three healthy controls for proteomics.

Age/Sex	Diagnosis	AQP-4
25/F	RRMS	ND
28/F	RRMS	ND
29/F	RRMS	ND
18/F	headache	-
35/F	headache	-
20/F	headache	-

## Data Availability

All data produced for this manuscript are available from the lead contact (Y.B.) upon reasonable request.

## Supplementary Materials

The following supporting information can be downloaded at: https://www.mdpi.com/article/10.3390/ijms26094160/s1.

## Abbreviations

CNS	central nervous system
DAPI	4′,6-diamidino-2-phenylindole
EAE	experimental autoimmune encephalomyelitis
GFAP	glial fibrillary acid protein
Iba-1	ionized calcium-binding adapter molecule 1
MBP	myelin basic protein
MOG	myelin oligodendrocyte glycoprotein
MS	multiple sclerosis
OLs	oligodendrocytes
OPC	oligodendrocyte progenitor cell
PBS	phosphate-buffered saline
PFA	paraformaldehyde
