# Supplementary material for: Implications of Chitinase 3-like 1 Protein in the Pathogenesis of Multiple Sclerosis in Autopsied Brains and a Murine Model
Source: Int J Mol Sci. 2025 Apr 27;26(9):4160. doi: 10.3390/ijms26094160 (PMC12071615; doi:10.3390/ijms26094160)
Supplement: Supplementary file 1 [file ijms-26-04160-s001.zip › ijms-3512213-supplementary.pdf]

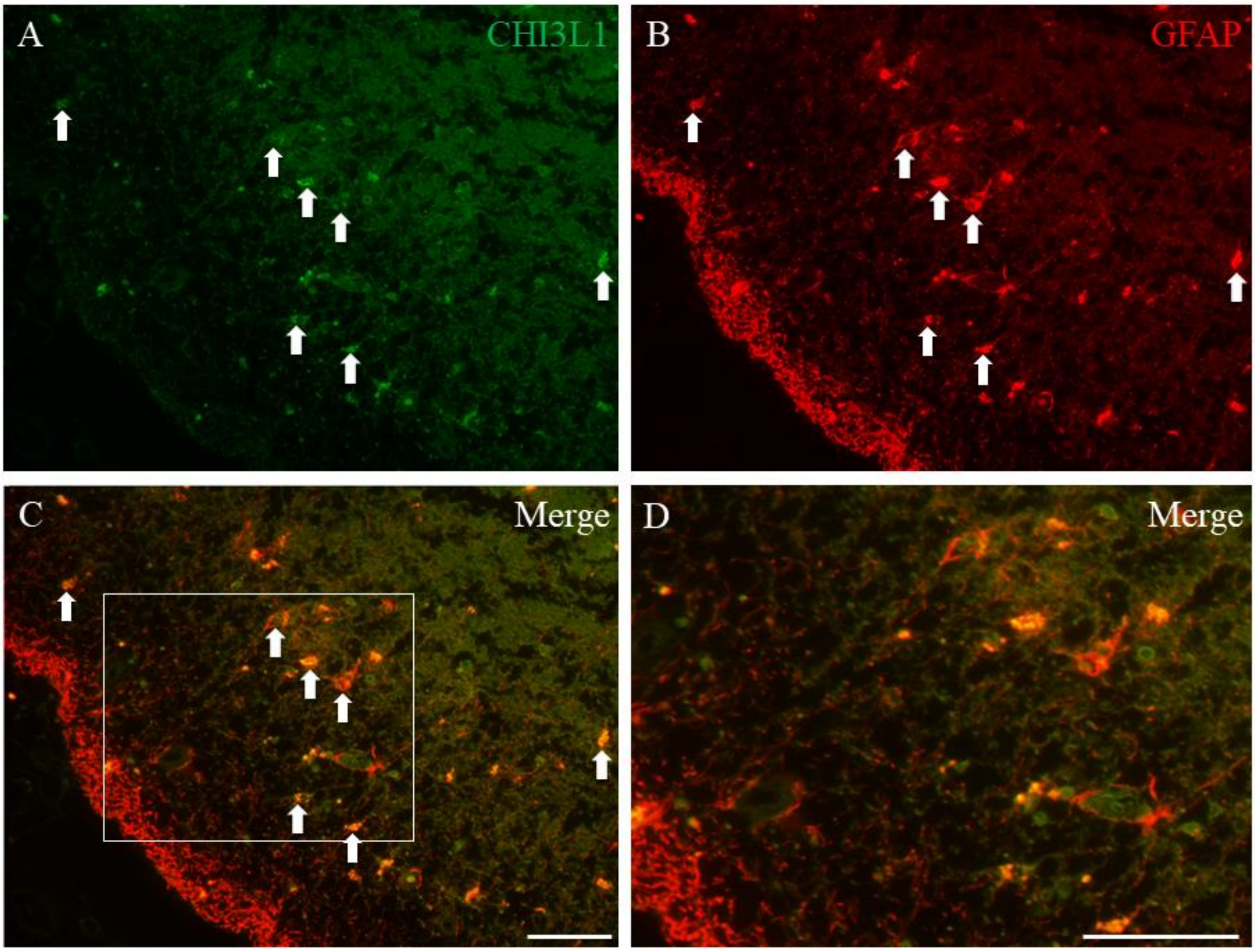

Supplementary Figure S1. Immunohistochemistry investigation in human autopsied MS brain. CHI3L1 expression in human autopsy specimens was examined using fluorescent double staining. Each panels show CHI3L1 (A, green), GFAP (B, red, an astrocytic marker), merge (C). High-power magnification of the square in panel C is also shown (D). White arrows indicate CHI3L1-expressing astrocytes. Scar bar = 50μm.
